# What is tongue-tie and does it interfere with breast-feeding? – a brief review

**DOI:** 10.3389/fped.2023.1086942

**Published:** 2023-04-25

**Authors:** Stephen M. Borowitz

**Affiliations:** Division of Pediatric Gastroenterology, Hepatology and Nutrition, University of Virginia, Charlottesville, VA, United States

**Keywords:** ankyloglossia, tongue-tie, infant feeding, breast-feeding, frenotomy, frenulotomy, frenectomy

## Abstract

The most common symptom attributed to ankyloglossia is difficulty breast feeding due to poor latch, inefficient milk extraction and/or maternal nipple pain. During the past two decades, despite a declining birth rate, there has been a dramatic increase in the number of infants diagnosed with and treated for ankyloglossia in the United States, Canada and Australia. Despite a dramatic increase in the diagnosis and treatment of ankyloglossia in these countries, there remains no universally agreed upon definition of ankyloglossia and none of the published scoring systems have been rigorously validated. However ankyloglossia is defined, the majority of infants with ankyloglossia are asymptomatic. Perhaps, infants with ankyloglossia have a greater incidence of difficulty breast feeding. Lingual frenulotomy may decrease maternal pain and at least transiently improve the quality of breast feeding in some infants however no published studies take into account the fact that sucking and feeding are soothing to infants and the observed improvements immediately following frenulotomy may be a response to the pain associated with the procedure rather than a result of the procedure itself. While there are almost certainly some infants in whom tongue-tie interferes with breast-feeding, there is currently no good evidence lingual frenulotomy leads to longer duration of breast-feeding. Frenulotomy appears to be a generally safe procedure however there are reports of serious complications. Finally, there are no studies of long-term outcomes following frenulotomy during infancy and given traditional thinking that the lingual frenulum is a cord of connective tissue tethering the tongue to the floor of the mouth may be incorrect and the frenulum contains motor and sensory branches of the lingual nerve, the procedure may be less benign than previously thought.

## Introduction

The most common symptom attributed to ankyloglossia is difficulty breast feeding, however some authors have asserted growth failure, aerophagia, gastroesophageal reflux and colic can be the result of ankyloglossia (1, 2). In older children, articulation problems, dysphagia, dental problems, sleep disordered breathing, migraine headaches and back and neck pain have all been attributed to ankyloglossia. Having said this, the only symptom for which there are any supporting data is difficulties breast feeding as a result of poor latch, inefficient milk extraction and/or maternal nipple pain (3).

Over the past 20 years, in the United States, there has been a dramatic increase in the number of infants diagnosed and treated for ankyloglossia despite a decline in the birth rate during the same period. Walsh and colleagues found between 1996 and 2012 there was a seven fold increase in the diagnosis of ankyloglossia and a ten-fold increase in the number of lingual frenulotomies performed (4). There has been a similar trend in Canada; between 2002 and 2014, the number of diagnosed cases of ankyloglossia rose from 7 to 23 per 1,000 births with a parallel increase in the number of frenulotomies performed (5). The same pattern has been seen in the state of Victoria in Australia where the frequency of frenulotomies rose from 1.6 per thousand children less than four years of age in 2006 to 5.04 per thousand children less than four years of age in 2016 (6). These data almost certainly under-represent the number of frenulotomies being performed as none of these data sets capture procedures performed by dentists.

This dramatic rise in the diagnosis of tongue tie and the number of frenulotomies being performed has not been universal. There has been no similar rise in Japan or in a number of countries in Europe including Italy, the Netherlands and the Scandinavian countries which have among the highest incidence of breast-feeding in the developed world (7).

## What is tongue tie?

Historically, the lingual frenulum has been considered a discrete cord or band of connective tissue with an anterioposterior orientation that tethers the tongue to the floor of the mouth. When the tongue is relaxed, the frenulum lies on the floor of the mouth, and when the tongue is elevated, the frenulum lifts with it (8). The general consensus is that tongue tie is “an embryological remnant of connective tissue underneath the tongue that failed to recede by apoptosis and that adversely impacts tongue function” (9).

Despite the dramatic increase in the diagnosis and surgical treatment of tongue tie in the U.S., Canada and Australia, there is no gold standard for to make the diagnosis of ankyloglossia. In many studies, the diagnosis is made “by eye”. There are however a number of different published scoring systems based upon the anatomy and/or function of the tongue and lingual frenulum.

One of the most widely used scoring systems is the Coryllos system published in 2004 in an American Academy of Pediatrics newsletter (10). The author does not explain how she developed her scoring system, how many infants she examined, or how the scoring system was validated and there are no published studies describing intra or inter-rater reliability (10).

Another widely used classification system is the Kotlow scoring system which was first published in 1999 (11). Dr. Kotlow was a pediatric dentist who developed his classification system by examining 322 children all of whom were at least 18 months of age. No information is provided on how he developed the system, or how it was validated. Dr. Kotlow revised his system in 2011 to include posterior tongue tie however no data are presented and the author spends much of the paper discussing the utility of laser therapy for ankyloglossia (12).

There are several published scoring systems incorporating the anatomy of the frenulum as well as tongue function and movement. The most widely cited is the Hazelbaker Assessment Tool for Lingual Frenulum Function. This scoring system was published in 1993 as a Master's thesis at Pacific Oaks College in California (13). The thesis is not available in the public domain and so it is unclear how many infants Dr. Hazelbaker used to develop her tool or how it was validated.

The Lingual Frenulum Protocol for Infants was published in 2013 and is comprised of an anatomic assessment and evaluation of nutritive and non-nutritive sucking (14). This tool was developed by a speech language pathologist in Brazil after examining 100 term infants and was validated by “two specialists” who viewed videotapes of her assessments. There is no published validation of this scoring system against any other assessment tools nor any published information regarding inter-rater reliability.

The British Tongue Assessment Tool (BTAT), published in 2015 is the most widely used assessment tool in the published literature (15). It was derived from evaluations of 224 term infants all of whom had difficulties breast-feeding. There were no control infants included in the tool's derivation. The validity of the BTAT has been measured against the Hazelbaker Assessment Tool based upon 126 assessments and the authors demonstrated good inter-rater reliability amongst the tool's developers. The TABBY tongue assessment tool is a simple picture version of the BTAT that was validated against the BTAT by five midwives who performed assessments on 262 infants referred due to ankyloglossia and breast-feeding difficulties (16).

The derivation of all these classification systems was based upon small numbers of infants the overwhelming majority of whom had difficulties breast-feeding. Moreover, recent anatomic studies have demonstrated that the lingual frenulum is not a fibrous band or cord, but rather, a fold of fascia that is in continuity from the floor of the mouth to the bottom of the tongue. The fold varies as the tongue moves. The frenulum is visually more prominent when the mucosa and fascia attach higher on the ventral tongue surface, closer to the tip, and the frenulum appears much thicker when some of the genioglossus is pulled up between the folds. These studies also identified branches of the lingual nerve extending from the frenulum onto the superficial ventral surface of the tongue that have direct connections to the motor end plates of intrinsic tongue muscles and thus likely have a direct role in shaping the contour of the tongue in response to sensory input (8, 17). These authors also point out that the lingual frenulum is not a band or string of fibrous tissue and so there is no anatomic basis for “posterior tongue tie”. They also point out the frenulum is sensate, and pain severe enough to require analgesia has been reported for as long as a week after frenulotomy in older children and adults.

Given the lack of a single agreed upon means of making the diagnosis of ankyloglossia, any estimate of its prevalence must be taken with a grain of salt. Estimates across the world vary between 0.1% and 15% of infants depending on the population studied and the criteria used. Most papers cite a prevalence of between five and ten percent (18). A number of studies suggest tongue tie is roughly twice as common in boys than girls, is more common in first-born infants, and there may be a family predilection.

## Is ankyloglossia associated with difficulties with breast feeding?

In May of 2020, the author performed a search of PubMed using the keywords ankyloglossia, tongue-tie, breast-feeding, breast milk, and infant feeding. Five studies were identified that prospectively evaluated whether ankyloglossia, however it was defined, was associated with difficulties breast feeding.

In a study conducted at Stanford University (19), healthy term newborns underwent an oral examination. Determination and grading of ankyloglossia were subjective. Of 1,041 infants, 50 or just less than 5% were diagnosed with ankyloglossia. 50 control infants were matched on factors thought to influence breast-feeding. The researchers made monthly phone calls asking mothers how breast feeding was going. Thirty-six tongue tied and 36 control infants completed the study (72%). At two months of age, 30 of 36 infants with ankyloglossia were still nursing, three of whom had undergone frenulotomy thus 82% of infants with untreated ankyloglossia were still breastfeeding at two months as compared to 91% of the controls. At the two month follow-up, eight mothers of infants with ankyloglossia reported nipple pain with feeds as compared to seven controls.

In a Brazilian study, 499 term newborns whose mothers were planning to breast-feed underwent oral examinations (20). Assessments were performed using the Lingual Frenulum Evaluation Protocol and the Bristol Tongue Assessment Tool and breast-feeding was assessed using the Unicef Breastfeeding and Observation Protocol. Fourteen or 3.1% of infants were diagnosed with ankyloglossia, three of whom were deemed severe and underwent frenulotomy during the first week of life. 9% of infants had poor breast-feeding assessment scores, most often due to abnormalities of mother's nipples. Of 14 infants diagnosed with ankyloglossia, three had difficulties nursing during the first week of life and underwent frenulotomy. The remaining 11 had no difficulties nursing and were still nursing at a month of age. Eighty percent of mothers of infants with ankyloglossia deemed the quality of breastfeeding good, and the remaining 20% fair while 92% of the mothers in the control group deemed feeding good, 7% fair, and 1% poor. All 11 infants with ankyloglossia who did not undergo frenulotomy were still breastfeeding at a month of age.

In a study from Israel, 200 term newborns were evaluated for ankyloglossia using the Coryllos classification system (21). The assessors were blinded as to whether the infant was having any difficulties nursing. After the examination, mothers completed a structured questionnaire on the quality of feeding, and a follow-up phone interview was conducted two weeks later. Thirty-eight percent of the infants were diagnosed with tongue tie. There was no association between tongue tie and mother reporting difficulties nursing and none of the infants with a tongue tie where the frenulum attaches to the tip of the tongue had difficulties nursing. Similarly, the authors found no association between the type of lingual frenulum and maternal pain with nursing.

In a study performed in Minnesota, nurses performed an oral exam on every newborn and if they suspected tongue tie, an investigator performed the Hazelbaker Assessment Tool and the mother completed an assessment of breast feeding competence (22). For every infant diagnosed with ankyloglossia, investigators identified two breast-fed infants without ankyloglossia as controls. One hundred forty-eight or 4.2% of infants were diagnosed with ankyloglossia. Forty-five infants with ankyloglossia were enrolled, and 38 completed the study. At a week, more of the infants with ankyloglossia were exclusively bottle fed compared to controls, however at a month, more of the control group were exclusively bottle fed. There were no differences between infants with ankyloglossia and controls in how well the mother thought breast feeding was going, how much pain they had while nursing, maternal concerns about growth, and whether the baby was always hungry.

In a study from Germany, a single investigator examined healthy term infants before they left the nursery using the Hazelbaker Assessment Tool (23). The principal outcome was mother's perceptions of breast feeding. Of 776 infants, 116 or 15% were diagnosed with tongue tie. Fifty-five percent of the infants with ankyloglossia had difficulties breast feeding as compared to 42% of infants without tongue-tie. The authors noted “a lack of breast-feeding experience was the highest risk factor for breast feeding problems” with an odds ratio of 4.4.

One of the problems with all these studies is that mother and family were not blinded to the investigator's findings, and it seems likely being told your infant is tongue tied might cause anxiety and influence how breast feeding goes. Nevertheless, based on these data, the majority of infants who are diagnosed with ankyloglossia do not appear to have difficulties breast-feeding, however there is a group of infants who do, and it raises the question of whether there is any causality between ankyloglossia and difficulties breast feeding and one way to try and answer this question is to release the tongue tie via frenulotomy and see if it helps.

## Does frenulotomy improve breast feeding?

Frenulotomy is performed by a wide-range of practitioners including midwives, lactation consults, physicians and dentists and this procedure is generally considered pain and risk-free. Historically, midwives performed a frenulotomy using a sharp fingernail, however currently most frenulotomies are performed with a scissors or scalpel without sedation or anesthesia, however there has been a recent trend of using a laser (24–26).

In April of 2023, the author performed a search of PubMed using the keywords frenulotomy, frenectomy, frenotomy, ankyloglossia, tongue-tie, breast-feeding, breast milk, and infant feeding. Five prospective studies were identified that compared frenulotomy to no frenulotomy or freunulotomy to a sham procedure and assessed the impact of the procedure on breast-feeding.

In a study of 60 infants between 5 and 115 days of age who had difficulties breast feeding and were diagnosed with ankyloglossia, Berry and colleagues randomized infants to frenulotomy or a sham procedure (27). Breast feeding was assessed by the mother and an observer before and immediately after the procedure. The mother and observer were blinded as to whether the infant had undergone frenulotomy. All infants randomized to the sham procedure underwent frenulotomy before they left the hospital and follow-up was conducted via telephone one day and three months after discharge. No information is included as to how ankyloglossia was diagnosed. While follow-up was conducted at one day and three months, all infants had undergone frenulotomies at the time of follow-up and so there wasn't any true control group. Mothers were more likely to report improvement in breast-feeding than were observers following both frenulotomy and sham procedure however according to mothers and observers, nearly half of infants who underwent sham procedure were breast-feeding better. While the amount of pain mothers reported decreased more with frenulotomy than with sham procedure, the differences were not statistically significant. The investigators also asked mothers and observers whether the infant had undergone frenotomy or not, and despite being blinded, 65% of mothers guessed correctly.

In a study of 58 infants between 1 and 35 days of age who had difficulties breast-feeding and had been diagnosed with ankyloglossia using the Hazelbaker Assessment Tool, Buryk and colleagues randomized infants to frenulotomy or sham procedure (28). Mothers were blinded until after the post-procedure assessment. Assessments of nipple pain and effectiveness of breast-feeding were performed immediately after frenulotomy or sham procedure and again two weeks later. The investigators did not determine how successful blinding was.

For infants who underwent a frenulotomy or sham procedure, pain scores decreased immediately after the procedure and decreased even further at two weeks, however the group that underwent frenotomy improved more at both time points. Breast feeding effectiveness improved significantly immediately after procedure in infants that underwent frenulotomy and were unchanged in infants that underwent a sham procedure, however at two week follow-up, scores between the two groups were identical. At two week follow-up, families in the sham group were offered frenulotomy, and 27 of 28 opted for the procedure, and so while the authors conducted follow-ups at two, six and twelve months, all but one of these infants had undergone frenulotomy.

In a study from Bristol, UK, 107 infants between 8 and 16 days of age with ankyloglossia and difficulties breast feeding were randomized to frenulotomy or routine breast-feeding support (29). Ankyloglossia was diagnosed using the Hazelbaker Assessment Tool and five days after randomization, breast-feeding efficacy and maternal pain with nursing were identical between the infants who had undergone frenulotomy and those that had not. The authors tracked was how likely mothers were to continue breast-feeding and there were no differences between the two groups at baseline or five days after randomization.

In a study from Southampton, UK, all infants born at four birthing centers were examined for ankyloglossia before leaving the hospital and mothers were monitored weekly to assess how breast feeding was going (30). Of 1,866 infants, 201 (11%) were diagnosed with ankyloglossia “by eye” and mothers were informed of the findings. Of these 201 infants 88 (44%) had difficulties feeding in the first month of life, 57 of whom participated in this study. Participants were randomized to frenulotomy or 48 h of intensive breast-feeding support. Average age at enrollment was 20 days. The principal outcome was mother's assessment as to whether breast-feeding had improved. Of the controls, one mother reported improvement at 48 h whereas 27 of 28 mothers whose babies had undergone frenulotomy reported improvement. 48 h after enrollment, mothers randomized to the control group were offered frenulotomy, and 27 of 28 opted for surgery. The authors state 27 of 28 experienced improvement, the overwhelming majority immediately after the procedure.

In a final study, 25 healthy term infants with ankyloglossia who had been referred to a lactation clinic because mother was experiencing pain with nursing were randomized to frenulotomy followed by a sham procedure or vice versa (31). Ankyloglossia was diagnosed by visual inspection. Feeding effectiveness and pain were determined during the first feeding immediately after the sham procedure or frenulotomy. Mothers were blinded as to the order of procedures, and observers watched to make sure nobody examined the infant's mouth immediately after the procedure. Frenulotomy was associated with small but statistically significant improvements in maternal pain and feeding effectiveness however the authors do not report data following sham procedures nor do they provide any other follow-up information.

So, does frenulotomy lessen difficulties associated with breast-feeding? The data we have just reviewed are not very compelling. The Cochrane Collaborative completed a review on this topic in 2017 and the five trials just reviewed are the only published papers that met their inclusion criteria (32). There are many published case series and a single retrospective cohort series which espouse the benefits of frenulotomy most of which have been published in the breast-feeding, ENT and dentistry literature, but none of these studies contain control groups of any kind. All five of the studies reviewed above have significant methodological shortcomings. The total number of infants included in these five trials is only 302. Only two studies blinded both mothers and assessors and all of the studies offered frenulotomy to controls and most controls opted to undergo the procedure. Perhaps most importantly, none of these studies reported whether frenulotomy led to long-term breast feeding success. Having said this, it is difficult to perform extended placebo controlled trials as if difficulties breasfeeding persist, families are likely to seek some form of therapy as evidenced by the fact that in several of the studies cited, the majority of infants in the sham or placebo group ultimately underwent frenulotomy. The authors of this review concluded “Frenotomy reduced breastfeeding mothers’ nipple pain in the short term. Investigators did not find a consistent positive effect on infant breastfeeding. Researchers reported no serious complications, but the total number of infants studied was small. The small number of trials along with methodological shortcomings limits the certainty of these findings”.

The Canadian Agency for Drugs and Technologies in Health conducted a systemic review in June of 2016 and concluded “Altogether, given the minimal harms and probable benefit, albeit of uncertain magnitude, frenectomy may be a viable treatment option for infants of mothers who wish to breastfeed and are experiencing difficulty” (33). They go on to say “The evidence underlying these conclusions comes primarily from poor-quality NRSs, and does not adequately address the question of whether frenectomy provides a meaningful incremental benefit over other treatments or procedures to improve breastfeeding, particularly in the long-term. Many potential confounders that could have contributed to variation in the observed outcomes were not controlled for”.

While no serious complications were reported among the 302 infants in the five trials cited above, and no serious complications were described among 237 infants who underwent laser frenulotomy in the largest published series of this procedure (26), there are numerous reports in the literature describing serious complications following frenulotomy. There are reports of delay in the diagnosis of other more serious causes of poor feeding such as congenital heart disease or metabolic disorders (34). Severe bleeding and hemorrhagic shock have also been reported (35, 36). There are also reports of post-procedure submandibular abscess, Ludwig's angina, apnea, acute life-threatening events and long term oral aversion/feeding refusal (37, 38). The New Zealand Pediatric Surveillance Unit prospectively surveyed pediatricians in New Zealand over a two year period and identified 23 serious complications following frenulotomy and they estimated this translates to moderate or severe complications in roughly 1% of infants undergoing the procedure (39). In an email survey sent to physician and dentist members of the Academy of Breastfeeding Medicine, 62% of 211 respondents reported caring for an infant whose feeding difficulties had been incorrectly attributed to ankyloglossia or had experienced a complication from frenulotomy (40).

## Conclusions

In conclusion, there is no clearly agreed upon definition of ankyloglossia and none of the published scoring systems have been rigorously validated. The traditional thinking that the lingual frenulum is a cord of connective tissue tethering the tongue to the floor of the mouth appears to be incorrect; the frenulum is a fold of fascia in continuity from the floor of the mouth to the bottom of the tongue and it contains motor and sensory branches of the lingual nerve and as a result there is no clear anatomic basis for a “posterior” tongue tie, a view shared by some members of the American Society of Pediatric Otolaryngology (41).

However ankyloglossia is defined, the majority of infants with ankyloglossia are asymptomatic. Perhaps, infants with ankyloglossia have a greater incidence of difficulty breast feeding. No research to date has identified specific characteristics of the lingual frenulum that clearly correlate with biomechanical dysfunction of the tongue, maternal pain during nursing and/or ineffective milk removal from mother's breast. Lingual frenulotomy may decrease maternal pain and at least transiently improve the quality of breast feeding in some infants however there is currently no evidence lingual frenulotomy leads to longer duration of breast-feeding. Having said this, it is important to acknowledge that it is extremely difficult to extend randomized trials of frenulotomy beyond the immediate post-procedure period as controls will likely seek additional treatment if difficulties breastfeeding persist.

None of the published studies on the impact of frenulotomy take into account the fact that sucking and feeding are soothing to infants and as such, the observed improvements immediately following frenulotomy may be a response to the pain associated with the procedure rather than a result of the procedure itself (42, 43). It has been shown that non-nutritive sucking attenuates the pain associated with circumcision (43) and in the author's experience, infants feed vigorously immediately after circumcision.

Frenulotomy appears to be a generally safe procedure however there are reports of serious complications. Far and away the most common complication is minor bleeding, estimated to occur in approximately 1% of cases, however there are numerous reports of much more serious complications. There are no studies of long-term outcomes following frenulotomy during infancy and given recent findings that the lingual nerve extends from the frenulum onto the ventral surface of the tongue, frenulotomy may compromise tongue sensation and movement, the procedure may be less benign than previous thought.

With these caveats, it seems likely that there are some infants in whom ankyloglossia interferes with successful breast-feeding by causing maternal nipple pain during nursing and/or by interfering with efficient milk transfer. In recent position papers, both the Academy of Breast-Feeding Medicine and the American Society of Pediatric Otolaryngology contend that among infants diagnosed with ankyloglossia, if maternal nipple pain and/or poor milk transfer cannot be remedied with appropriate conservative measures, a frenulotomy can be offered (41, 44).
